# From protein-protein interactions to immune modulation: Therapeutic prospects of targeting Neuropilin-1 in high-grade glioma

**DOI:** 10.3389/fimmu.2022.958620

**Published:** 2022-09-20

**Authors:** Gregory T. Smith, Daniel P. Radin, Stella E. Tsirka

**Affiliations:** ^1^ Molecular and Cellular Pharmacology Graduate Program, Department of Pharmacological Sciences, Renaissance School of Medicine at Stony Brook University, Stony Brook, NY, United States; ^2^ Stony Brook Medical Scientist Training Program, Department of Pharmacological Sciences, Renaissance School of Medicine at Stony Brook University, Stony Brook, NY, United States

**Keywords:** Neuropilin-1 (NRP1), high grade glioma (HGG), glioma-associated macrophages and microglia, hypoxia, angiogenesis, cytotoxic T cell, glioma, Treg cells

## Abstract

In the past several years there has been a marked increase in our understanding of the pathophysiological hallmarks of glioblastoma development and progression, with specific respect to the contribution of the glioma tumor microenvironment to the rapid progression and treatment resistance of high-grade gliomas. Despite these strides, standard of care therapy still only targets rapidly dividing tumor cells in the glioma, and does little to curb the pro-tumorigenic functions of non-cancerous cells entrenched in the glioma microenvironment. This tumor promoting environment as well as the heterogeneity of high-grade gliomas contribute to the poor prognosis of this malignancy. The interaction of non-malignant cells in the microenvironment with the tumor cells accentuate phenotypes such as rapid proliferation or immunosuppression, so therapeutically modulating one target expressed on one cell type may be insufficient to restrain these rapidly developing neoplasias. With this in mind, identifying a target expressed on multiple cell types and understanding how it governs tumor-promoting functions in each cell type may have great utility in better managing this disease. Herein, we review the physiology and pathological effects of Neuropilin-1, a transmembrane co-receptor which mediates signal transduction pathways when associated with multiple other receptors. We discuss its effects on the properties of endothelial cells and on immune cell types within gliomas including glioma-associated macrophages, microglia, cytotoxic T cells and T regulatory cells. We also consider its effects when elaborated on the surface of tumor cells with respect to proliferation, stemness and treatment resistance, and review attempts to target Neuroplin-1 in the clinical setting.

## Introduction

Our understanding of tumor cell intrinsic and microenvironmental factors driving the genesis, treatment resistance and recurrence of high-grade gliomas has drastically increased in recent years. Such insight notwithstanding, the standard of care for glioblastoma (GBM) remains maximal safe surgical resection coupled with radiation and chemotherapy with the methylating agent temozolomide (1). Since 2005, the only addition to the standard of care for newly diagnosed glioblastomas has been the Optune tumor-treating fields (TTF) therapy, which extended median patient survival from 16 to approximately 20 months (2). Regardless, this aggressive multimodal treatment regimen yields a dismal 5-year survival rate of about 10% (1).

It is worth noting that the approved therapies for GBM, excluding surgical resection, all seek to target rapidly cycling tumor cells. These treatments may artificially select for cells resistant to genotoxic stressors (3, 4), and tumor cells may elicit adaptive resistance cues from cells in the tumor microenvironment (TME) (5–13). Evolution of glioma cells after treatment may explain why some therapies exhibit efficacy in newly diagnosed gliomas but lack effectiveness in the recurrent setting (14, 15). To overcome the challenges to decidedly target tumor cells, it became important to understand the manner in which non-cancerous cells in the TME contribute to the progression, treatment resistance and recurrence of high-grade gliomas. Such research has been fruitful, as we now understand that cells like tumor-associated neurons, OPCs, astrocytes, pericytes, microglia and macrophages all contribute to the pathogenesis of high-grade gliomas (16). Targeting paracrine interactions may be useful, as they would be specific to the tumor area and avoid side effects commonly associated with DNA damaging agents.

Studies on resected tumors show that microglia and macrophages can make up ~30% of the cells in a glioma biopsy (7, 9–12, 17–23). As a corollary, we now appreciate that the degree of infiltration of glioma-associated microglia/macrophages (GAM) correlates with glioma invasiveness and glioma grade (6, 24, 25). Therefore, of all the non-cancerous cells in the TME, a considerable emphasis has been placed on understanding the molecular mechanisms driving the oncogenic crosstalk between glioma cells and GAM, in order to identify druggable nodes that can be leveraged for clinical benefit. Our laboratory studies the role macrophages and microglia play in the progression of glioma. Prior work demonstrated that Neuropilin-1 (Nrp1), a co-receptor expressed on the surface of microglia and macrophages, sustains a robust neoangiogenic program as well as an immunosuppressive tumor microenvironment (21, 22, 26, 27). Deletion of Nrp1 from both myeloid cell types or from one of these cell populations mitigates tumor growth by slowing angiogenesis and relieving the immunosuppressive nature of the glioma TME. Additionally, pharmacological antagonism of the b1 domain, the domain through which transforming growth factor beta (TGFβ) and vascular endothelial growth factor (VEGF) exert the effects, mimics the functional outcomes of GAM-Nrp1 deletion which highlights that this protein may be a viable therapeutic candidate (21, 22, 26).

In addition to our studies examining the role of GAM-elaborated Nrp1, several other reports have been published looking at the role of Nrp1 in the context of other immune cells, endothelial cells, as well as the role of Nrp1 in tumor cell migration, treatment resistance, stemness, and response to ionizing radiation. Herein, we review the work describing how Nrp1 impacts on the function of multiple immune cells of the TME and on endothelial cells. The role of Nrp1 on tumor cells with respect to stemness, radiation resistance and targeted treatment resistance will also be considered. Moreover, we assess the contexts in which small molecule Nrp1 antagonists may be useful and compare them to the functional effects achieved by protein ablation or knockdown through RNA interface (RNAi). Finally, clinical attempts to antagonize Nrp1 will be reviewed as well as directions for future research.

## Nrp1 in endothelial cell physiology

**Figure 1:**
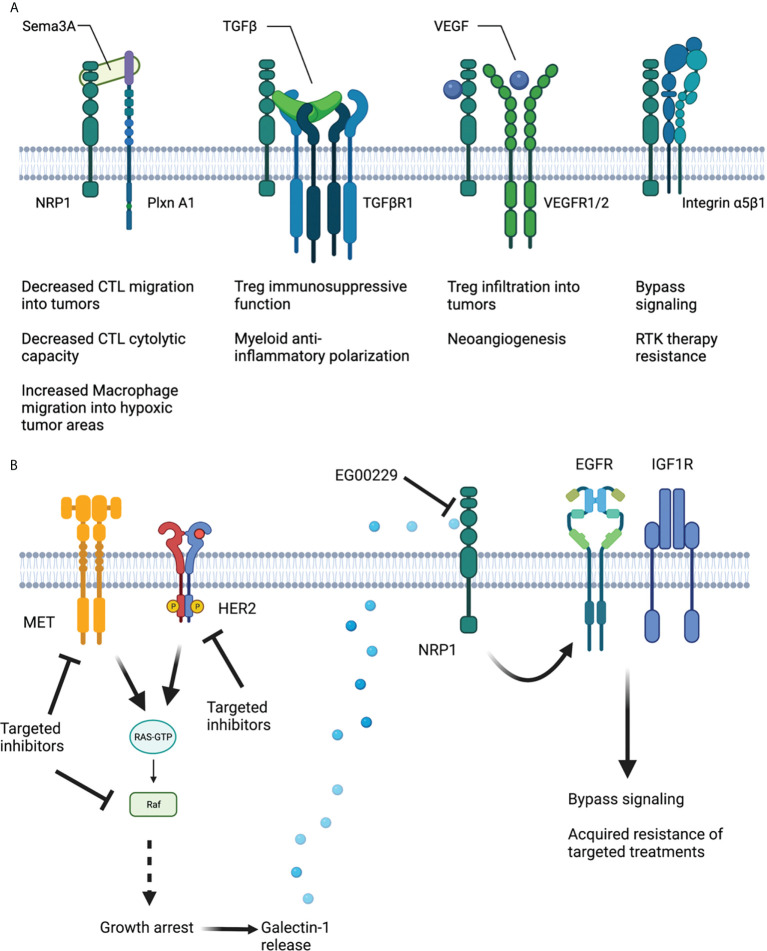
Schematic of NRP1 structure and interaction with various co-receptors to modulate immune cell function and targeted treatment resistance. **(A)** Sema3A hinders cytotoxic T cell migration into tumors and their cytolytic capability. SEMA2a also encourages macrophage migration into hypoxic tumor areas to resolve hypoxia and accelerate tumor progression. TGFβ augments Treg immunosuppressive function and myeloid anti-inflammatory polarization in a NRP1-dependent fashion. VEGF, binding to NRP1 and VEGFR1/2 increases Treg infiltration into tumors and promotes neoangiogenesis. NRP1 along with Integrin α5β1 encourages bypass signaling to support adaptive resistance to RTK therapies. **(B)** Therapies designed to inhibit MET, Her2 and BRAF signaling in tumor cells result in Galectin-1 release in a manner amenable to NRP1 b1 domain inhibition. Ligation of Galectin-1 to NRP1 results in EGFR and IGF1R activation and subsequent bypass signaling and acquired adaptive treatment resistance. Created using Biorender.com.

Nrp1 is a vascular endothelial growth factor (VEGF) co-receptor that is implicated in angiogenesis in the tumor microenvironment (28–30). As a co-receptor, NRP1 governs and modulates the signaling activity of several diverse cell surface receptors in many difference cell types (**Figure 1**).

Nrp1 was first associated with embryonic vascular development when modulation in endothelial Nrp1 expression led to widespread vascular defects including hemorrhagic and leaky blood vessels (31, 32). Multiple groups detailed Nrp1 loss of function effects, including embryonic lethality associated with severe vascular deficits underpinned by unbranched and underdeveloped blood vessels (32, 33). The microenvironment of the neurovascular unit, including pericytes, astrocytes, fibrocytes, and neurons, is in a complex interplay with the endothelial cells forming the vasculature (34). Antagonism of Nrp1 may perturb the neurovascular unit and provide information on the local function of Nrp1.

**Figure 2:**
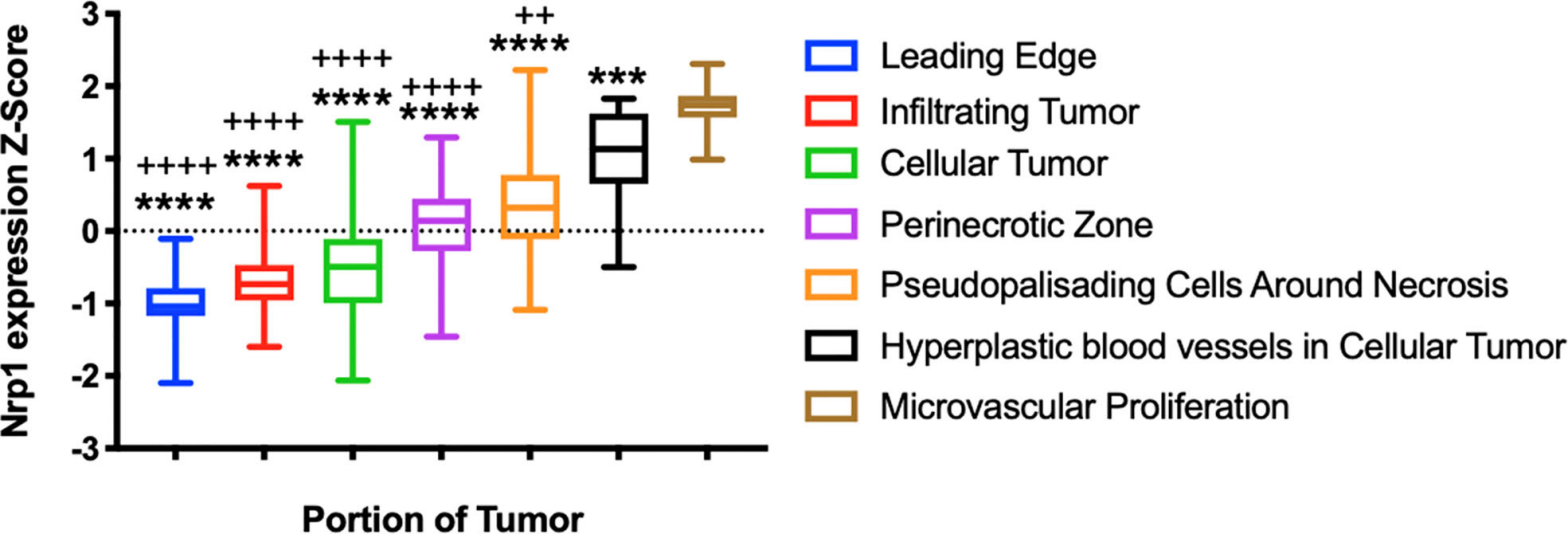
Neuropilin-1 in Glioblastoma. A) Relative expression of Nrp1 across glioblastoma tumor areas. ****p < 0.0001 ANOVA, demonstrating significant differences in expression across glioblastoma tumor areas. ++p < 0.01, ++++p < 0.0001, Brown-Forsythe test compared to Nrp1 expression in hyperplastic blood vessels. ***p < 0.001, ****p < 0.0001, Brown-Forsythe test compared to Nrp1 expression in areas of microvascular proliferation. Analysis from the Ivy Glioblastoma Atlas Project data (https://glioblastoma.alleninstitute.org/).

Nrp1 expression has been associated with increased tumor angiogenesis through The Cancer Genome Atlas (TCGA) analysis. Nrp1 expression was increased in primary tumors when compared to normal tissue, including head and neck squamous cell carcinoma, renal clear cell carcinoma, thyroid carcinoma, stomach adenocarcinoma, and liver hepatocellular carcinoma. Nrp1 overexpression was associated with a reduction in the endothelial markers [e.g., Platelet Endothelial Cell Adhesion Molecule-1 (Pecam-1), Angiogenin, Phosphatidylinositol Glycan Anchor Biosynthesis Class F (PIGF) and matrix metalloproteinase-9 (MMP-9)], cytokines and chemokines [e.g., interleukins IL-6, IL-8, IL-1B, IL-4, transforming growth factor β3 (TGF-β3) and chemokine (C-C motif) ligand 2 (CCL2)]. Cytokines, chemokines and their receptors have been associated with promoting angiogenesis and the homing of circulating endothelial progenitor cells to sites of arterial injury. On the other hand, other C-X-C chemokines have been shown to inhibit neovascularization (35). Additional factors, such as mitogen-activated protein kinase-7 (MAPK7), tropomyosin 1 (TPM1), ribosome-binding protein 1 (RRBP1), protein tyrosine phosphatase receptor type K (PTPRK), heat shock protein 90A (HSP90A), RG24 and osteonectin/secreted protein acidic and rich in cysteine (SPARC), also normally associated with neoangiogenesis, were modulated by Nrp1 overexpression. The effects were dependent on semaphorin 4D (SEMA4D). These observations led to the conclusion that angiogenesis promoted by NRP1 results in a reduction of endothelial cell maturity (36). This term describes the transition from dependence on growth factors to the formation of stable vessel walls, partial destruction of the microvasculature, evident pericyte coverage and the presence of basement membrane (37). Anatomical annotations of relative Nrp1 expression in glioblastomas (**Figure 2**) illustrated that its expression was detected specifically in areas of hyperplastic blood vessels and areas of microvascular proliferation (38).

Nrp-1 induces SEMA4D-mediated increase in angiogenesis and *in vitro* tube forming activity. Their experiments revealed that overexpression of Nrp1 significantly increased the expression of Sema4D, cell migration and angiogenesis. Knock-down of Sema4D decreased the expression of Nrp1 at baseline levels in the inferior mesenteric artery vascular endothelial cell line (Ealy929), but had a smaller effect when Nrp1 was overexpressed. Sema4D silencing significantly reduced Ealy929 cell migration and the angiogenic index.

In endothelial cell lines and cells [Ealy929, microvascular endothelial cells (MVECs), primary human umbilical vein endothelial cells (HUVEC)] Nrp1 overexpression was shown to result in significantly increased cell proliferation, cell migration and angiogenesis. When Nrp-1 was knocked down in the cells, significantly lower rates of proliferation, migration and angiogenesis were observed. Interestingly, Nrp1 overexpression bypassed the effects of Sema4D knockdown, bringing cell migration and angiogenesis back to baseline, suggesting that Nrp1 overexpression and resulting signaling can circumvent the need for ligand binding (36).

### Biophysical properties

In a more biochemical and cell focused study, Nrp1 was associated with the formation of ternary complexes with vascular endothelial growth factor A (VEGFA) and vascular endothelial growth factor receptor 2 (VEFGR2), which resulted in increased endothelial cell migration through increased VEGFR2 activity. The experiments of Morin etal. (39) examined three states involved with Nrp1 complexes: *cis*, *trans* and a combination. In the “*cis*” state Nrp1 and VEGR2 are expressed on the same endothelial cell. The “*trans*” state involves two different cells, a tumor cell (expressing Nrp1) and an endothelial cell (expressing VEGFR2). The combination of cis and trans states describe expression of VEGFR2 on endothelial cells, while NRP1 is present on both endothelial and tumor cells (which is the most likely scenario). Interestingly, the *trans* state resulted in decreased tumor angiogenesis which may then lead to suppressed tumor initiation. The study, examining Nrp1 and VEGFR2 interactions in pancreatic ductal adenocarcinoma (PDAC) patients, revealed that the *trans* configuration of Nrp1 could lead to lack of engagement of endothelial VEGFR2 through possible disrupted signaling pathways, such as in extracellular regulated kinase (ERK). This *trans* localization of NRP1 with VEGFR2 ultimately suppresses tumor angiogenesis, reduces proliferation, and promotes patient survival (39). Thus, the downregulation of Nrp1 on endothelial cells may hold promising therapeutic potential.

Honing in on the VEGFR2-Nrp1 axis, King etal. (40) studied the homo-interactions of Nrp1 along with the hetero-interactions of Nrp1 and VEGFR2 (40). VEGFR2, a regulator of endothelial proliferation and migration, dimerizes and is activated when vascular endothelial growth factor A (VEGFA) binds to each subunit. VEGFA binds to Nrp1 as well and further activated VEGFR2. VEGF-165 or VEGFA-165 is a secreted isoform of VEGFA and can bind to both VEGFR2 and Nrp1 (41). Using Fluorescence resonance energy transfer (FRET) methods, NRP1 was shown to associate with VEGFR2 in both the presence and absence of the ligand VEGFA, thus supporting a ligand-independent interaction (40, 42). The association with VEGFR2 presence may lead to a decrease in NRP1 self-oligomerization. In the absence of the VEGFA-165 ligand, the dimeric fraction of NRP1 increased. The presence of the VEGFA-165 ligand resulted in an increase in NRP1 oligomer formation (40). At high NRP1 cell surface density, VEGFA-165 results in the reduction of oligomeric NRP1 molecules, as the balance shifts toward an increase of the number of Nrp1 molecules associated with the Nrp1/VEGFR2 complex. Thus, increasing ligand leads to a rearrangement of the complex. Addition of VEGFR2 to the NRP1 oligomers in the absence of VEGFA-165 leads to a rearrangement of the oligomeric complex, possibly through an increase in VEGFR2-NRP1 interactions. The binding interactions of NRP1 with VEGFR2 are an important consideration when designing therapeutics, as compounds similar to VEGFA-165 may affect NRP1/VEGFR2 complex formation and the resulting intracellular signaling.

### Nrp1 in the neurovascular environment

Nrp1 has been investigated for its role in the neuroinflammatory-neurovascular axis in an autoimmune encephalomyelitis (EAE) model (43). Knockout of the endothelial Nrp1 was found to reduce EAE disease progression in a murine model with a decrease of C-X-C motif chemokine ligand 10 (CXCL10) and interferon-γ (IFN-γ) levels which was dependent on Rac1 (Ras-related C3 botulinum toxin substrate 1). Specifically, knockdown of Nrp1 in human brain microvascular endothelial cells (HBMVECs) suppressed the IFN-γ-mediated activation of signal transducer and activator of transcription 3 (STAT3) and CXCL10 through Rac1 signaling. Moreover, compared to WT controls of EAE, the endothelial Nrp1 knock-out mice displayed an intact blood-brain-barrier (BBB) and preserved tight junctions. A heterozygous deletion of Nrp1 also resulted in intact BBB integrity visualized through FITC-labeled albumin leakage analysis. When the neuro-immune environment was assessed in the EAE model, while there were no differences in the CD4^+^CD8^-^, CD4^+^CD8^+^, or CD4^-^CD8^-^ T cells infiltrating the spinal cord, but there was an observed reduction in natural killer T cells in the Nrp1 knockout mice (43).

The effects of NRP1 on the neurovascular unit are critical in the context of gliomas and glioblastomas. The actual presence of intact BBB (composed of continuous endothelial cells connected by intercellular tight and adherens junctions, a basement membrane, pericytes, microglia and perivascular astrocyte end-feet) in glioblastoma patients has been debated. While in many cases the compromise of BBB has been verified using contrast dynamic contrast enhanced (DCE)-magnetic resonance imaging (MRI), not all tumor areas exhibit such compromise, and indeed some tumor areas are devoid of contrast. This becomes significant for drug delivery, since areas with intact BBB are not readily accessible by chemotherapeutics that are not BBB permeable (44). VEGF, induced by local tumoral hypoxia, drives the disruption of BBB and the formation of altered and more permeable new capillaries (45). The blood-brain-tumor-barrier (BBTB) was examined in a series of studies, and the hyperpermeability of the tumor capillaries to fluorescently labeled albumin was associated with the number of pores present was assessed: in average-size tumor about 30% of the vessels had fenestrations and about 10% had open junctions (46). When the maximal fenestration size was investigated in RG-2 glioma using different size nanoparticles, it was shown that the fenestrations are up to 250 nm, but the therapeutically relevant upper limit of BBTB pore size is approximately 11.7 to 11.9 nm (47).

The consequences of the endothelial Nrp1 knockout from Wang etal. (43) are in agreement with the subsequent work from Morin etal. (39) which indicates that the endothelial Nrp1 is a key molecule of interest in disease models, as Nrp1 overexpression on endothelia may be associated with increased migration, proliferation, and angiogenesis (39). These neovascular implications may worsen tumor progression through alterations in the Nrp1/VEGFR2 complex arrangements, expression of pro-inflammatory cytokines, and a reduction in endothelial maturation factors which would otherwise result in a static phenotype. Endothelial Nrp1 remains a key target of interest as its inhibition may result in therapeutic advances. However, considerations should be maintained regarding the plasma membrane complex formations and rearrangements from the addition of such an antagonist.

## Nrp1 in neuronal physiology

Beyond endothelial signaling, NRP1 is heavily involved in neurodevelopment, neuronal survival and migration, and axon guidance (48). VEGFR2 binds to a PlexinD1/NRP1 receptor complex in neurons, forming a trimeric receptor complex for semaphorin 3A (SEMA3A) (49). Upon SEMA3A binding, tyrosine residues in the complex become phosphorylated, thereby activating a phosphatidylinositol-3 kinase/Ak strain transforming protein (PI3K/Akt) pathway. Subsequent activation of this pathway ultimately may result in axon growth.

During development, NRP1 is implicated in commissural axon guidance through VEGF signaling (50). Using an mRNA-miRNA functional analyses, Nrp1 mRNA was shown to be upregulated along with roundabout guidance receptor 1 (Robo1), EPH receptor A3A (Epha3), Unc-5 netrin receptor C (Unc5c), DCC Netrin 1 receptor (Dcc), P21 Rac1 Activated Kinase 3 (PAK3), and Lim kinase-4 (Limk4) in mature neurons associated with facilitating axon guidance (51). As the retinal ganglion cell (RGC) axons either avoid or cross the midline at the optic chiasm, VEGF164 signals through Nrp1 on contralateral RGC axons to promote chemoattraction during this process, thereby promoting contralateral growth (50).

SEMA3A signals through NRP1 and 2 to guide gonadotropin-releasing hormone (GnRH) neurons in the developing brain (52). Moreover, VEGF-164 acted as a survival cue for migrating GnRH neurons through ERK/Akt signaling, mediated by the Nrp1 neuronal receptor independently from VEGFR2. Specifically, an immortalized GnRN cell line, GN11, was used to study the role of VEGF-164 and Nrp1 on cell survival using serum withdrawal experiments. VEGF-164 administration prolonged cell survival after 24hrs periods of serum withdrawal. However, the pro-survival effects of VEGF-164 were reversed when Nrp1 was blocked using a blocking antibody. In these experiments, VEGFR2 was not detectable in GN11 cells with RT-PCR; further Cre-induced loss of VEGFR2 in the survival experiments support the idea that NRP1 mediates cell survival independently of VEGFR2. Similar experiments revealed that VEGF-164/Nrp1 signaling activated PI3K/AKT/MAPK pathways leading to increased cell survival in GnRH neurons (52).

In the context of a glioma, new emphasis has been placed on understanding the roles that neurons and neuron-secreted and tumor-secreted axon guidance molecules may play in tumor development. Netrin is one of these molecules associated with tumor invasiveness as it was shown to change non-invasive GBM cells into invading ones and lead to increased expression of GBM stem-like cell markers (53). Similarly, and relevant to NRP1, SEMA3A inhibited glioma-stem cell proliferation and stimulated invasion in a NRP1- and PlexinA1-dependent manner (54). NRP1 was also shown to function as a receptor for glial-derived neurotrophic factor (GDNF) in GBM, promoting proliferation of the glioma cells (55).

### Neuronal hypoxia studies

During oxygen glucose deprivation (OGD), cultured rat cortical neurons were found to upregulate Nrp1 and Sem3A when compared to controls. However, inhibition of Nrp1 with an antagonist resulted in protection from OGD-induced cell death. It may be possible that expression of Sema3A/Nrp1 results in inhibition of axon regeneration following injury. However, there appeared to be a protective effect of Sema3A transfection in neurons exposed to similar OGD treatments (56). Thus, inhibition of Nrp1 may have protective effects in areas of hypoxia, which may be of utmost importance in an area of tumor cells as hypoxia regions are frequently present. However, considerations should be made regarding inhibition of Nrp1 with axon migration in normoxic conditions; although this may not be applicable in an otherwise developed brain.

### Nrp1 in immunity; an overview of immuno-therapeutic relevance

Nrp1 is evolving as a target of interest within the realm of neuro-immunity, as its inhibition appears to be a potential therapeutic avenue with clinical promise (21). Elevated Nrp1 expression in microglia and macrophages have been linked with a poor prognosis in gliomas (27). Specifically through Kaplan-Meier survival analysis, differences between NRP1 low and NRP1 high populations were observed both in low grade and in high grade gliomas. In the low grade cohort, the NRP1 Low median survival was 114 months, while in the NRP1 High the median survival was 62.91 months (p val = 0.036). In the GBM Cohort. the patients with low NRP1 had a median survival of 13.76 months, while those with high NRP1 had a median survival of 10.42 (p val = 0.040). When both low and high grade glioma patients were combined to represent glioma of all grades, NRP1 Low median survival was 114 months, whereas the NRP1 High median survival was 15.93 months (27).

### Nrp1 in microglia and macrophages

Microglia and macrophages comprise between 30% and 50% of a glioma tumor (57), (58). It has been previously found that microglial ablation in models of high grade glioma results in reduced glioma growth (57), (59). Possible mechanisms involve glioma-associated microglia and macrophages (GAMs) releasing pro-tumorigenic cytokines such as TGFβ, EGF, Il-6, and IL-1β (57) (59) while microglia alone may upregulate platelet-derived growth factor receptor (PDGFR) in both murine and human low- and high-grade glioma (57), (60). Specifically, M2-polarized microglia induce upregulation of PDGFR in glioma cells, which lead to an increase in glioma migratory capacity (60). These cell types, along with monocytes, have an associated increase in Iba1 and CD11b, respectively, across human glioblastoma classifications (27, 61). Corroborating this study, Nrp1 expression was shown to be significantly correlated with the expression of ionized calcium binding adaptor molecule 1 (Iba1) and CD11b in a glioblastoma patient cohort (27).

To investigate Nrp1 functionally our laboratory assessed the localization of Iba1with Nrp1 in from glioma I-IV patient biopsy specimens, revealing an infiltrate of Nrp1^+^Iba1^+^ cell types, GAMs. Follow up genetic ablation of Nrp1 in microglia and macrophages phenocopy results seen by pharmacological inhibition of Nrp1, which all resulted in a decrease in tumor volume and vascularity. Interestingly, Nrp1 ablation in these murine models of glioma resulted in an increase of Nrp1^-^Iba1^+^ GAMs near the tumor border. These tumor border GAMs also contained an altered immune phenotype; specifically, there was a shift towards an anti-tumorigenic phenotype, indicated by an increase in the classically activated: alternatively activated (M1:M2) ratio, assessed by CD86:CD206 ratios (21). The increase in M1:M2 ratio of the GAMs is accompanied by impaired activation of the suppressor of mothers against decapentaplegic 2/3 (SMAD2/3) pathway, evident using an Nrp1 agonist, tuftsin (the tetrapeptide TKPR, which shares homology with the C-terminal domain of VEGF and was originally described as an exclusive agonist for Nrp1 (62, 63). Tuftsin activation of Nrp1 results in an M2, anti-inflammatory, pro-tumorigenic, and pro-phagocytotic phenotype in microglia through the canonical TGFβ signaling cascade with a consequential increase in SMAD2/3 signaling. This M2 phenotype could be reversed with a Nrp1 antagonist, EG00229, resulting in an anti-tumorigenic M1 polarization (63). We utilized these compounds for *in vitro* assessment of SMAD2/3 following Nrp1 inhibition and activation in WT and Nrp1-KO microglial cell lines in the presence and absence of glioma associated cytokines (21). No differences were found between WT and Nrp1-KO microglia at basal states; however, WT microglia showed an increase in SMAD2/3 activation compared to no changes in Nrp1-KO microglia when treated with GL261 glioma-conditioned medium. Pre-treatment with EG00229 revealed similar results: WT microglia pre-treated with the Nrp1 inhibitor did not show an increase in SMAD2/3 signaling after treatment with glioma cytokines (21).

### Nrp1 in GAMs & hypoxia

High degrees of hypoxia remain a feature of grade IV glioma (64). In the context of glioma, the cell proliferation can initially lead to regions depraved of vasculature. In response, hypoxic cells release VEGF to begin neovascularization mediated through HIF1α. While the neovascularization may be initially thought to counteract the low oxygen partial pressures, the opposite may be true. Specifically, the hypoxia-induced neovascularization may result in abhorrent, occluded vessel formation (64) (65). We recently observed that inoculating mice with syngeneic GL261 glioma stem-like cells resulted in robust tumors after 3 weeks with a markedly disorganized tumor vasculature: blood vessels had minimal patency and a small overall luminal area (66, 67). The consequence of the formation of this new vasculature includes maintenance of low oxygen partial pressures and selection for aggressive, hypoxic cancer cells (64, 66).

In 2013 Casazza et al. investigated the role of Nrp1 in the trafficking of macrophages into hypoxic areas (26). The group found that hypoxia induced Sema3A-mediated chemoattraction for tumor-associated macrophages through the VEGFR/Nrp1/PlexinA1/PlexinA4 receptor complex. Conversely, mice lacking Nrp1 in macrophages (LysM-Cre;Nrp1^L/L^) exhibited reduced tumor progression in models of non-CNS carcinomas: specifically, the loss of Nrp1 in macrophages resulted in 55% fewer lung metastases and 60% smaller tumors. While proliferation remained unchanged in LysM-Cre;Nrp1^L/L^ mice, apoptosis increased. The group further showed that the vessel area in the tumor, vessel perfusion, vessel branching points, and vessel density were decreased when macrophages lacked Nrp1. The investigators further reported that tumor associated macrophages lacking Nrp1 did not traffic into hypoxic areas. Interestingly, there were nearly twice as many myeloid cells infiltrating into the tumor in the LysM-Cre;Nrp1^L/L^ mice compared to controls, potentially due to an increase in tumor hypoxia and reduced tumor perfusion. Interestingly, the hypoxia attractants, including colony stimulating factor 1 and 2 (Csf1 and Csf2), and CCl2, were higher in LysM-Cre;Nrp1^L/L^ mice at the end of the experiment; however, the LysM-Cre;Nrp1^L/L^ tumor associated macrophages were mainly found in areas of normoxia and absent in areas of hypoxia (26). With regard to the expression of Nrp1 in hypoxia and normoxia, Casazza etal. (26) reported that Nrp1 is transcriptionally repressed in a hypoxic environment. These studies were conducted with bone marrow-derived macrophages (BMDMs) and isolated tumor associated macrophages, where Nrp1 was reduced by 80% and 90%, respectively. The transcriptional response was reversed when I kappa B kinase beta (IκBKβ) was deleted from macrophages. One possible mechanism could be the prevention of forming an I kappa B kinase (IκK) complex to activate the canonical nuclear factor kappa light chain enhancer of activated B cells (NF-κB) pathway, thereby repressing Nrp1 (68). However, the repression was restored when p50/p65 NF-kB subunits were overexpressed in both IKBKB-KO and hypoxia inducible factor 2α -knock out (HIF2α-KO) macrophages. Therefore, HIF2α induction leads to Nrp1 transcriptional repression through IκK-mediated activation of the canonical NF-κB pathway, achieved through activation of p50/p65 heterodimer (26). Honing further into the signaling responses, the group found that WT BMDMs migration doubled when the cells were exposed to SEMA3A while Nrp1-KO macrophages did not respond (26). Similarly, VEGF164 led to a reduction in Nrp1-KO macrophage migration by 30% when compared to WT controls. However, VEGF treatment still resulted in a response marked by phosphorylation of VEGFR1. Thus, there appeared to be a Nrp1 dependency on the action of Sema3A in the migratory response. Loss of macrophage Nrp1 also significantly reduced Sema3A-dependent VEGFR1 activation, yet knockdown of VEGFR1 in both WT and Nrp1-KO macrophages halted migration in both SEMA3A and VEGF164 treatment. Migration was also halted when PlexinA1 and PlexinA4 were silenced during VEGF164 and SEMA3A treatment in Nrp1-KO macrophages. Thus, the presence or absence of Nrp1 can lead to activated migratory signaling cascades or halted migration, respectively, at least on macrophages (26). Investigating the spatial positioning of the tumor associated macrophages with knock-out and WT macrophages, the group reported that Sema3A drives tumor associated macrophage trafficking into areas of hypoxia in a Nrp1-dependent manner. Upon entry into the hypoxic niche, Nrp1 is downregulated, thereby reducing Sema3A-mediated migratory responses, and trapping the tumor associated macrophages in the hypoxic niche (26). Overall, these data suggest that Sema3A attracts macrophages through a VEGFR1/NRP1 complex.

## Nrp1 in T Lymphocytes

### Nrp1 in CD4^+^ T lymphocytes

Nrp1 is a marker for the CD4^+^Foxp3^+^ (forkhead box P3) regulatory T cells (T_regs_), the solid tumor-infiltrating CD8^+^ T cells, and a subset of CD4^+^ T cells (69). In 2002, Tordjman et al. discovered that Nrp1 interactions initiate the primary immune response in human T cell and dendritic cell (DC) populations; specifically, Nrp1 mediated contacts between DCs and T cell clusters and facilitated resting T cell proliferation. When Nrp1 was blocked with antibodies, the DC-mediated proliferation of resting T cells was inhibited by 60% and 50%, respectively, when DCs and T cells were preincubated with Nrp1 blocking antibodies. However, this preincubation had no effect on the proliferation of activated T cells. Since this discovery, investigations have been launched into the role of Nrp1 on T cell functioning within the context of tumor biology.

Bruder etal. (70) corroborated the findings of Tordjman etal. (69) by reporting that anti-Nrp1 treatment inhibited murine CD4^+^ T cell activation (70). However, the suppressor function of CD4^+^CD25^+^ T cells were not affected by anti-Nrp1 preincubation. Thus, the group sought to assess the suppressor activity of CD4^+^Nrp^High^ and CD4^+^Nrp^Low^ T cells on CD4^+^CD25^-^ and CD4^+^CD25^+^ T cells, with the latter being used as a control. The results from this study revealed that only CD4^+^Nrp^High^ T cells suppressed the proliferation of naive CD4^+^CD25^-^ T cells (69, 70). Moreover, Nrp1 expression paralleled FoxP3 expression in naive, regulatory, and activated CD4^+^ T cell populations. FoxP3 is an established marker of CD4^+^CD25^+^ T_reg_ (71), (72). Ectopic expression of Foxp3 in CD4^+^CD25^-^ T cells resulted in increased Nrp1 expression, and shifting to a T_reg_ phenotype (70).

In cancer, infiltrating CD4^+^Foxp3^+^ T cells have been associated with suppression of an anti-tumoral response. In murine melanoma models, it was discovered that CD4^+^Foxp3^+^ T cells migrate to tumor areas through Nrp1 receptor signaling associated with VEGF ligands (73). Ablation of Nrp1 in T cells led to significantly reduced tumor volume when compared to WT controls. It was further found that populations of CD4^+^ T cells were significantly reduced in Nrp1-KO models when compared to controls. Interestingly, peritumoral CD8^+^ T cell populations remained stable and unchanged yet intratumoral CD8^+^ T cell populations increased with Nrp1 ablation. Thus, T cell-specific ablation of Nrp1 led to an increase in the anti-tumoral, intratumoral CD8+ T cell population while decreasing tumoral CD4^+^ T cell populations (73). There were no differences in the suppressive activity of Nrp1-deficient and WT CD4^+^Foxp3^+^ T cells, indicating that Nrp1 does not act in this capacity. Nrp1-KO tumors were also characterized by elevated TGFβ and Sema3A mRNA compared to controls. Moreover, WT CD4^+^CD25^+^ Treg cells expressing Nrp1 were found to migrate towards VEGF, whereas Nrp1KO CD4^+^CD25^+^ Treg cells lacked the capacity to migrate towards VEGF. These data suggest that VEGF acts an attractant cue for CD4^+^Foxp3^+^ T cells and that tumor infiltration is facilitated by Nrp1 expression. Ablation of Nrp1 leads to an increase in anti-tumoral intratumoral CD8^+^ T cells (73).

Moreover, Nrp1 contributes to Treg stability through Sema4a interactions leading to inhibition of the phosphatase and TENsin homolog deleted on chromosome 10 (PTEN)/Akt/Foxo (forkhead family of transcription factors) axis (74). Upregulation of this pathway was suggested to promote T cell survival, especially in areas of inflammation. The group investigated a NRP1 mutant in which the cytoplasmic post synaptic density/drosophila disc large tumor suppressor/zonula occludens-1 proteins (PDZ) domain was deleted; under these conditions, Nrp1 failed to inhibit the phosphorylation of AKT at the immunological synapse and recruit PTEN. Upon AKT activation, Foxo transcription factors are phosphorylated and subsequently translocated and sequestered from the nucleus. Thus, under normal conditions, a NRP1-SEMA4a complex inhibits the phosphorylation of Akt through PTEN signaling and leads to nuclear translocation of Foxo transcription factors, thereby promoting Treg survival through activation of Foxo targets including Foxp3 (74).

High numbers of intratumoral Nrp1^+^ Tregs are associated with poor prognosis and outcomes in melanoma and head and neck squamous cell carcinoma (75), as they are driving a shift towards an IFNγ-insensitive immunosuppressive phenotype for CD8^+^ cells that is conducive to tumor growth. Conversely, Nrp1 deficiency leads to a fragile Treg phenotype with elevated intracellular HIF1α and IFNγ leading to lifting the suppression on CD8^+^ T cells. It is possible that the fragile T cell phenotype is conducive to anti-programmed death-1 protein (anti-PD1) therapy whereas the robust phenotype can be resistant to PD1 immunotherapy (75).

### Nrp1 in CD8^+^ T cells

CD8^+^ cytotoxic T cells have a crucial role in anti-tumor immunity and hold therapeutic promise (76). CD8^+^ T cells must traffic to the tumor and release cytotoxic granules and pro-inflammatory chemokines through T-cell receptor major histocompatibility complex-1 (TCR-MHC-1) mediated signaling (76). Investigating the role of Nrp1 and CD8^+^ T cell physiology further, Leclerc etal. (76) reported that Nrp1 may be an immune-checkpoint negative regulator of the CD8^+^ anti-tumor phenotype (76). Using models of human small cell lung carcinoma, the investigators found that a higher proportion of CD8^+^ cells, rather than CD4^+^ cells, were expressing Nrp1, and that SEMA3a-NRP1 interactions inhibit CD8^+^ cell functions. Migration studies revealed that T cell migration towards CXCL12 was inhibited when the cells were exposed to SEMA3a. Beyond migration, cytotoxic phenotypes were also impaired during activation of NRP1 by SEMA3a, thereby suggesting that the NRP1:SEMA3a axis is a negative regulator of a cytotoxic phenotype in CD8^+^ cells. When anti-Nrp1 monoclonal antibodies or anti-PD1 immunotherapy was used, alone or in combination, a larger infiltration of CD8^+^ cytotoxic T cells from combination therapy was reported, compared to either therapy alone. The combination therapy increased CD8^+^:CD4^+^ ratios and led to an increase in serine protease granzyme B, Ki67, and killer cell lectin like receptor G1 (KLRG1) in tumor infiltrating CD8^+^ cells. These markers are associated with cytotoxic activity, proliferation, and terminal differentiation in effector T cells, respectively. Similarly, tumor infiltrating CD8^+^ T cells from mice treated with the above combination therapy displayed higher efficacy in killing a colon cancer cell line, MC-38, most likely due to the increase in granzyme B expression (76).

Moreover, Nrp1 inhibition may restore an anti-tumoral state in T cells (77). While assessing biomarkers, Liu etal. (77) found that Nrp1^+^CD8^+^ tumor infiltrating lymphocytes (TIL) were characterized by increased proliferation (BrdU+ cells) and T cell activation markers, namely elevated CD44, CD69, and CD25. They also expressed lower levels of naive T cell markers, such as CD62L, CD127, and KLRG1 (77). Furthermore, a clinical trial is currently assessing the efficacy of anti-Nrp1 monoclonal antibody (ASP1948) therapy in combination with anti-PD-l therapy (Nivolumab/Pembrolizumab), expected to lead to Treg cell inhibition (NCT03565445) (78, 79). The effects of Nrp1 inhibition on CD8^+^ and Treg cells, or the possibility of using Nrp1 expression as a cancer biomarker make Nrp1 a promising candidate molecule for study (80).

### Nrp1, stemness and resistance

Stemness describes the ability of cells to self-renew and to generate differentiated cells (81). In cancer stemness poses challenges including cancer aggressiveness and resistance to therapy.

Beck etal. (81) discovered that Nrp1 is necessary for tumor initiation in models of skin papilloma and that deletion of Nrp1 in cutaneous cancer stem cells (CSCs) inhibited VEGF-mediated promotion of stemness. Using DMBA/TPA treatment to induce papillomas, Nrp1 deficient and control mice were compared in their ability to drive tumor initiation and progression. After 25 weeks of treatment, all control mice developed skin papillomas, but none of the Nrp1 deficient mice developed a neoplasm. A marker used to describe cancer cells stemness is CD34: Nrp1 was expressed the highest in CD34^+^ cells when compared to normal interfollicular keratocytes, hair follicle bulge stem cells, and CD34^-^ tumor epithelial cells. denoting a correlation of Nrp1 expression with cancer stemness. Probing the role of VEGF further, the group overexpressed VEGF while knocking out Nrp1; the results indicated tumor epithelial cells overexpressing VEGF resulted in accelerated tumor growth. However, when Nrp1 was knocked out in tumor epithelial cells with VEGF overexpression, the prior accelerated tumor growth was not observed. While tumor angiogenesis was still observed in Nrp1 knockout models, there was a lack of VEGF-mediated proliferation and no increase in CD34^+^ CSC population. These experiments indicate that the Nrp1-VEGF axis contributes to cancer cell stemness and tumor initiation. Interestingly, no changes in cell growth were observed when anti-Nrp1 prevented Sema interactions. This work unveiled the role of the Nrp1:VEGF axis in a stem cell niche where therapeutic intervention against Nrp1 holds the potential to reduce cancer stem cell stemness (81).

The interplay between VEGF, VEGFR2 and Nrp1 in the maintenance of glioma CSC and tumor progression was assessed (82). In comparing CD133- non-stem tumor cells (NSTC) and CD133+ CSC, CSC expressed higher levels of VEGFR2 on their cell surface (82). Highly VEGFR2-expressing CSC exhibit increased sphere formation and proliferation as well as increased VEGF secretion *in vitro*. Further, these cells exhibited increased tumorigenicity *in vivo*. However, these cells were amenable to shRNA-mediated knock down of both VEGFR2 and Nrp1. Knock down of both factors reduced proliferation and increased caspase-3/7 activity, respectively, *in vitro*. VEGFR2 knockdown also prolonged survival of glioma-bearing mice *in vivo*. Of particular note, VEGFR2 antagonism was superior to VEGF sequestration by bevacizumab in prolonging survival *in vivo* and enhancing response to radiation *in vitro*.

In a different study, VEGF-A was required for the stemness phenotype, not acting through VEGFR1/2, but rather through NRP1 (83). When Nrp1 was knocked down in models of human epidermal squamous cell carcinoma tumors, epithelial cancer stem cell spheroid formation was inhibited; tumor formation was further reduced and cancer cell migration and invasion was inhibited. qRT-PCR revealed upregulation of VEGF-A, Nrp1, and Hif1α in the epithelial cancer stem cell-derived tumors. Conversely, VEGFR1/2 were expressed at markedly reduced levels compared to that of Nrp1. Analysis with VEGF-A-siRNA and Nrp1-siRNA resulted in reduced cancer stem cell spheroid formation and migration whereas targeting VEGFR1/2 had no effect. Pharmacological inhibition with EG00229 resulted in a 25% reduction in invasion and significantly reduced tumor growth and vascularization (83).

**Figure 3:**
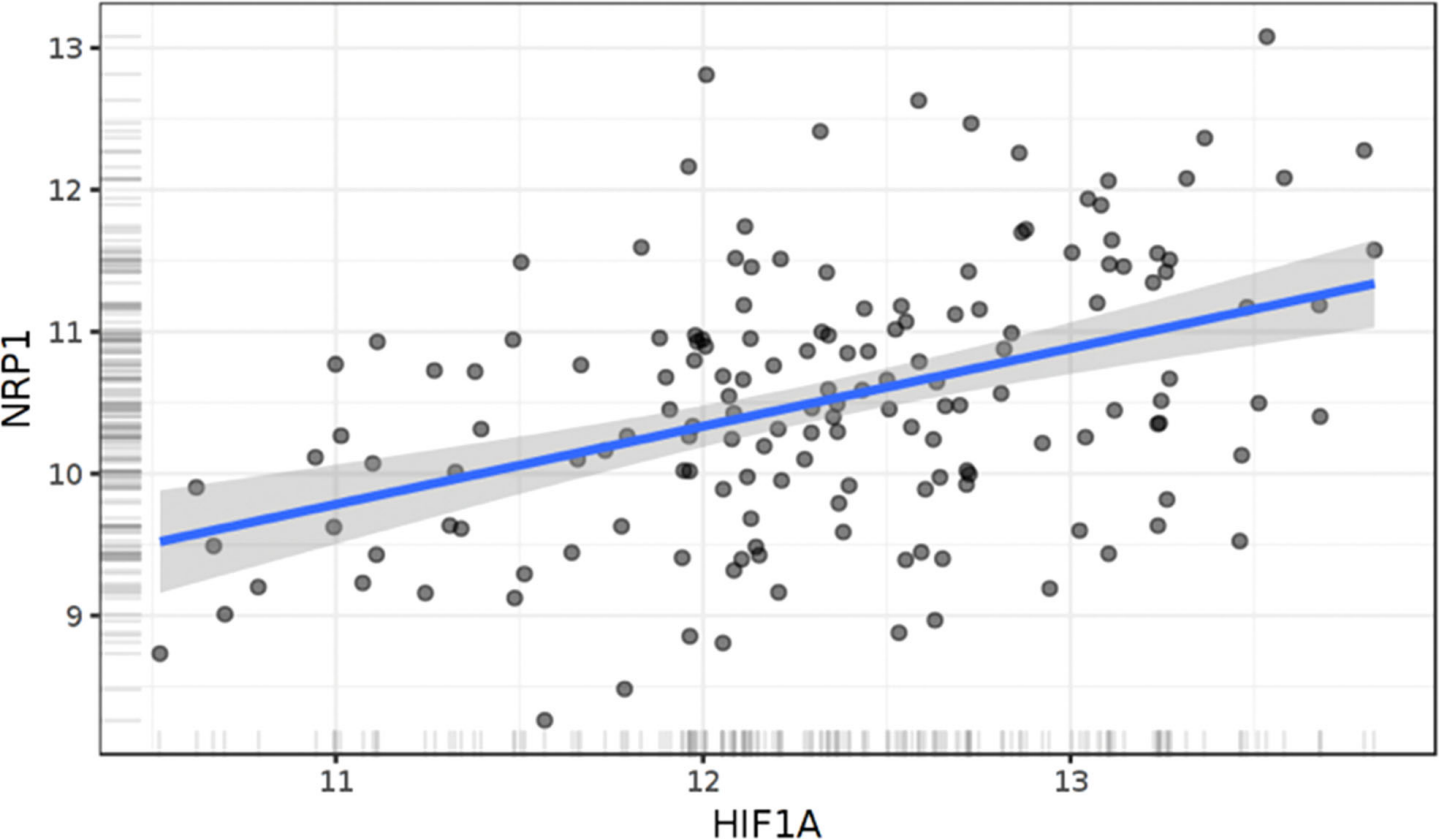
Correlation of Hif1α and Nrp1 expression in human glioblastoma. Linear regression of NRP1 expression against HIF1α. The results shown here are based upon data generated by the TCGA Research Network: https://www.cancer.gov/tcga." N=158 patients. R=0.42. p<0.0001, Pearson .

With specific respect to the tumor cell-specific interplay between Nrp1 and hypoxia, there is one study to our knowledge that has uncovered a specific interaction between Hif1a and Nrp1. Recently in models of lung adenocarcinoma, it was shown that Hif1a knock down also reduces Nrp1 levels in two NSCLC cell lines. The HIf1a stabilizer cobalt chloride, increased Hif1a protein levels as well as levels of Nrp1 (84). In their functional studies, HIf1a knock down reduced vasculogenic mimicry, tumor cell migration, invasion and would closure in a scratch assay. These effects were all reversed by Nrp1 overexpression. Using a luciferase reporter assay and chromatin immunoprecipitation, it was shown that HIf1a binds to the Nrp1 promoter and regulates Nrp1 levels. However, it is not known whether this also occurs in glioma cells, even though Hif1a levels correlate with Nrp1 levels in glioblastoma (**Figure 3**).

Interaction between Nrp1 and VEGF-A also mediated promotion of cancer cell stemness. In Michigan Cancer Foundation-7 (MCF-7) breast cancer cells, Nrp1-VEGF-A signals through wingless/integrated protein (Wnt)/β-catenin pathways, to confer stemness and chemoresistance (85). Activation of the Wnt/β-catenin signaling cascades have been previously associated with cancer cell invasion and motility (86). One study assessed the role of Wnt/β-catenin signaling in pediatric high-grade glioma cell lines and reported that antagonism of β-catenin/CBP (cyclic adenosine monophosphate response element binding protein) with ICG-001 resulted in reduction of cancer cell invasion and migration, as well as reduction in tumor sphere formation and proliferation (87). The RNA binding protein, Lin28B, was shown to bind to the 3’UTR of Nrp1 and stabilize the mRNA transcript, enhancing Nrp1 expression, and leading to an increase in Wnt/β-catenin signaling (88). These observations point to the broader conclusion that in VEGF-driven tumor cells where Nrp1 is highly expressed, pharmacological targeting of the b1 domain by small molecules, like EG00229, may be critical to curb tumor progression and limit CSC maintenance.

Nrp1 appears to signal through other pathways to confer stemness (89). For example, GSC-secreted Sema3C signals through a Nrp1/PlexinA2/PlexinD1 receptor complex to enhance sphere formation and GSC phenotype. Nrp1 may act as the ligand-binding receptor in the co-receptor complex with PlexinA2/PlexinD1. Experiments utilizing PlexinA2 and PlexinD1 knockdowns resulted in inhibition of Rac1 activity. While Nrp1 receptor knockdowns were not assessed, there may be an association between Sema3C -mediated glioma stemness through Nrp1/PlexinA2/PlexinD receptor binding and downstream Rac1 activation. Overall, these experiments supported the role of Rac1-mediated GSC tumorigenicity and survival through Sema3C signaling (89).

The role of Nrp1 in chemoresistance was also examined (90). While one avenue of targeting melanoma involved inhibiting the BRAF kinase, drug resistance ensued from BRAF-addicted cancer cells, mediated through downregulation of sex-determining region Y-related high mobility group box 10 protein (SOX10) *via* miRNA-338, which was associated with Nrp1 expression in melanoma (91). EGFR receptor expression was also similar and comparable with Nrp1 expression in the model. Gastric (GLT16) and lung cancer-derived cell lines (EBC1 and H1993) were employed and Nrp1 was downregulated by shRNA, which led to increased sensitivity to MET inhibitors. This suggested that Nrp1 mediates chemoresistance in v-raf murine sarcoma viral oncogene homolog B1 (BRAF-) and cMet-oncogene addicted cell lines. In a model of breast cancer, the same investigators examine Nrp1 levels during chemotherapy: in response to the human epidermal growth factor receptor 2 (HER2) inhibition with trastuzumab Nrp1 was increased. Similarly, the HER2 addicted cell line, SKBR3 and BT474, had higher Nrp1 expression with increasing chemoresistance to lapatinib. However, the mechanisms seen in the melanoma tumors were not observed across cancer models; for instance, SOX10 did not appear to mediate resistance in the HER2 addicted cells. Similarly, miR-338 was not affected in the breast cancer model. Thus, Nrp1 appears to promote chemoresistance in a variety of tumor models, yet the mechanisms behind resistance appear to be variable depending on the nature of the tumor (90). It is also important to note that EG00229 exhibited minimal effects on parental cells, but in cells that exhibited acquired resistance to targeted therapies, Nrp1 knock down and EG00229 treatment were sufficient to resensitize tumor cells to the targeted therapies (90). The mechanism by which Nrp1 is engaged in cells that had acquired resistance to targeted therapies seems to involve secretion of galectin-1 by treatment resistant tumor cells in an autocrine manner. Galectin 1 engages the b1 domain of Nrp1 and induces treatment resistance as a bypass signaling mechanism (92), suggesting that Nrp1 b1-domain targeting may be useful in tumors that secrete high levels of galectin-1.

## Conclusions and future directions

As a co-receptor associating with multiple receptors, NRP1 can modify the signaling activity taking place in many difference cell types (**Figure 1**). Such cells exhibit unique functions in the initiation, progression, treatment resistance or targeting of various tumor types. The domains of Nrp1 that are engaged in these activities critically determine functional outcomes and cellular responses, especially in a tumor microenvironment as complex as that fostered by high-grade gliomas. Targeting the a1 domain to perturb semaphorin 3A binding may reduce myeloid cell migration to hypoxic tumor areas and may offset their M2-polarization all while also enhancing the cytolytic function of cytotoxic T cells. Additionally, targeting the b1 domain of Nrp1 may compromise TGFβ and VEGF effects on myeloid cells, T cells and cancer stem cells, all of which are critical effectors in tumor progression, treatment resistance and tumor recurrence. The many functions of NRP1 in high-grade tumors are complex. Therefore, further efforts must be placed in determining the manner in which NRP1 can be targeted to offset its multiple pro-tumorigenic roles, potentially reconciling the significant pre-clinical data obtained when Nrp1 is knocked out or down using shRNAs. This may allow efficient targeting NRP1 in the clinical setting to compromise CSC and possibly as adjuvants to immunotherapies that have already exhibited efficacy in a subset of patients. Combinatorial approaches may allow to augment the efficacy of therapies like PD1/PD-L1 antagonists and induce long-lasting clinical responses and hinder tumor relapse.

## Author contributions

DR, GS and ST wrote drafts and edited the text. All authors contributed to the article and approved the submitted version.

## Funding

This work was partially supported by an NIH F30CA257677 (DPR), and a RSOM/Stony Brook University Walk for Beauty grant (SET).

## Acknowledgments

The authors would like to thank members of the Tsirka lab for thoughtful discussions.

## Conflict of interest

The remaining authors declare that the research was conducted in the absence of any commercial or financial relationships that could be construed as a potential conflict of interest.

The reviewer JN declared a past collaboration with the author ST to the handling editor.

## Publisher’s note

All claims expressed in this article are solely those of the authors and do not necessarily represent those of their affiliated organizations, or those of the publisher, the editors and the reviewers. Any product that may be evaluated in this article, or claim that may be made by its manufacturer, is not guaranteed or endorsed by the publisher.
